# Dosimetric benefits of intensity‐modulated radiotherapy and volumetric‐modulated arc therapy in the treatment of postoperative cervical cancer patients

**DOI:** 10.1002/acm2.12003

**Published:** 2016-11-21

**Authors:** Xia Deng, Ce Han, Shan Chen, Congying Xie, Jinling Yi, Yongqiang Zhou, Xiaomin Zheng, Zhenxiang Deng, Xiance Jin

**Affiliations:** ^1^ Radiotherapy and Chemotherapy Department the 1st Affiliated Hospital of Wenhzou Medical University Wenzhou China; ^2^ Department of Clinical Solutions Support Elekta Instrument (Shanghai) Ltd. Shanghai China

**Keywords:** cervical cancer, intensity‐modulated radiotherapy, volumetric‐modulated arc therapy, whole pelvic conformal radiotherapy

## Abstract

As the advantage of using complex volumetric‐modulated arc therapy (VMAT) in the treatment of gynecologic cancer has not yet been fully determined, the purpose of this study was to investigate the dosimetric advantages of VMAT by comparing directly with whole pelvic conformal radiotherapy (CRT) and intensity‐modulated radiotherapy (IMRT) in the treatment of 15 postoperative cervical cancer patients. Four‐field CRT, seven‐field IMRT, and two‐arc VMAT plans were generated for each patient with identical objective functions to achieve clinically acceptable dose distribution. Target coverage and OAR sparing differences were investigated through dose‐volume histogram (DVH) analysis. Nondosimtric differences between IMRT and VMAT were also compared. Target coverage presented by V95% were 88.9% ± 3.8%, 99.9% ± 0.07%, and 99.9% ± 0.1% for CRT, IMRT, and VMAT, respectively. Significant differences on conformal index (CI) and conformal number (CN) were observed with CIs of 0.37 ± 0.07, 0.55 ± 0.04, 0.61 ± 0.04, and CNs of 0.33 ± 0.06, 0.55 ± 0.04, 0.60 ± 0.04 for CRT, IMRT, and VMAT, respectively. IMRT and VMAT decreased the dose to bladder and rectum significantly compared with CRT. No significant differences on the Dmean, V45, and V30 of small bowel were observed among CRT, IMRT, and VMAT. However, VMAT (10.4 ± 4.8 vs. 19.8 ± 11.0, *P* = 0.004) and IMRT (12.3 ± 5.0 vs. 19.8 ± 11.0, *P* = 0.02) decreased V40, increased the Dmax of small bowel and the irradiation dose to femoral heads compared with CRT. VMAT irradiated less dose to bladder, rectum, small bowel and larger volume of health tissue with a lower dose (V5 and V10) compared with IMRT, although the differences were not statistical significant. In conclusion, VMAT and IMRT showed significant dosimetric advantages both on target coverage and OAR sparing compared with CRT in the treatment of postoperative cervical cancer. However, no significant difference between IMRT and VMAT was observed except for slightly better dose conformity, slightly less MU, and significant shorter delivery time achieved for VMAT.

## 1 Introduction

Cervical cancer is one of the most common gynecologic cancers worldwide with approximately 83% of the cases happened in the developing countries.^1^ Concurrent radiotherapy with cisplatin‐based chemotherapy has become the standard treatment for early stage cervical cancer patients with positive pelvic nodes and/ or positive surgical margin and/ or positive parametrium according to the National Comprehensive Cancer Network (NCCN) guidelines.^2^, ^3^ Postoperative whole pelvic conformal radiotherapy (CRT) has become the standard of care for patients meeting specific criteria thanks to multiple phase III trials showing the benefit of pelvic radiation on reducing the risk of pelvic recurrence in patients with a high‐risk pathologic feature.^4^, ^5^

The toxicity of conventional CRT is a result of the large volume of normal tissues irradiated, especially small bowel, rectum, bladder, and bone marrow. Conventional CRT using two or four photon fields result in the majority of the true pelvis receiving the prescription dose (usually 45–50 Gy in 25–28 fractions). After a hysterectomy, small bowel falls into the pelvis where the uterus previously resided, further increasing the amount of small bowel irradiated to prescription dose. Rates of grade 2 and higher acute gastrointestinal (GI) toxicity of 50–90% with conventional CRT have been reported in the literature.^6^ Acute GI symptoms typically involve varying degrees of diarrhea, cramping and abdominal pain, which can negatively impact quality of life during treatment.^7^

Over the last decade, interest in the use of IMRT to treat gynecologic cancer has been increasing. The IMRT technique has the potential benefit over conventional CRT of improving target coverage, reducing the volume of the organs at risk (OARs) that receive irradiation, and reducing the toxicity to normal tissue.^8^ Dosimetric studies have shown a significant reduction in the dose to small bowel with IMRT when compared to conventional CRT. Heron et al compared a seven‐field IMRT plan with four‐field box technique on ten consecutive patients referred for postoperative radiotherapy and showed a 52% reduction in the volume of small bowel receiving more than 30 Gy with the IMRT.^9^ A similar study by Roeske et al reported a 50% reduction in the volume of small bowel irradiated to more than 45 Gy with a nine‐field IMRT plan when compared with a conventional four‐field box technique in ten patients with either endometrial or cervix cancer.^10^ Portelance et al demonstrated a 58–67% reduction in the volume of small bowel receiving more than 45 Gy with IMRT when the number of fields used was increased from four to nine.^11^

Despite the significant benefits of IMRT, there are some disadvantages. The technique usually requires multiple fixed‐angle radiation beams, which can increase treatment delivery time and has an impact on patient comfort, reproducibility of the treatment position, and intra‐fraction motion. Moreover, IMRT uses a larger number of monitor units (MUs) compared with conventional CRT, leading to an increase in the amount of low‐dose radiation received by the rest of the body. This raises the concern of secondary radiation‐induced malignancy, which is of particular relevance to young patients or those with long future life expectancies.^12^, ^13^

Volumetric‐modulated arc therapy (VMAT) is an extended form of IMRT with variable dose rate, gantry speed, and dynamic multileaf collimator movement.^14^ VMAT plans with faster delivery time, fewer MU, and superior dose distribution than conventional step‐and‐shoot IMRT have been reported.^15^ With this capability of delivering a highly conformal dose distribution within a short time interval, VMAT has been widely accepted by the radiotherapy community. Cozzi et al compared the dosimetric difference between IMRT and RapidArc on eight cervix uteria cancer patients and observed both RapidArc and IMRT resulted in equivalent target coverage but RapidArc had an improved homogeneity and conformity index, as well as dose reduction on OARs.^16^ Sharfo et al. compared 9, 12, and 20 beam IMRT with single and dual arc VMAT for ten cervical cancer patients and indicated that 12 and 20 beam IMRT were superior to single and dual arc VMAT, with substantial variations in gain among the study patients. The author concluded that often reported increased plan quality for VMAT compared to IMRT has not been observed for cervical cancer.^17^

As we can see, advantage of using the complex VMAT techniques in the treatment of gynecologic cancer has not yet been determined.^18^, ^19^ The purpose of this study is to investigate the dosimetric advantages of VMAT by comparing directly with IMRT and 3D whole pelvic CRT in the treatment of 15 postoperative cervical cancer patients.

## 2 Materials and methods

### 2.A Patients and simulation

Fifteen consecutive patients with cervical cancer after hysterectomy were enrolled in this study. All of the patients had squamous cell carcinoma. Staging was performed according to the International Federation of Gynecology and Obstetrics (FIGO) classification. All patients were immobilized in the supine position using a thermoplastic abdominal fixation device. Computed tomography (CT) simulation was performed for each patient using a 16‐slice Brilliance Big Bore CT scanner (Philips Healthcare, Cleveland, OH.) with intravenous contrast. Contiguous 3‐mm slices were taken from the iliac crest to the ischial tuberosities. All CT datasets were transferred into a commercial treatment planning system (Monaco 5.1.1; Elekta, Crawley, UK) to design the plans.

### 2.B Contour and treatment planning

The clinical target volume (CTV) was contoured according to the consensus guideline of the Radiation Therapy Oncology Group (RTOG) 0418 and its atlas on the RTOG website, which comprises a central vaginal CTV and a regional nodal CTV.^20^ The former included the proximal vagina and paravaginal tissues and the latter consisted of the common iliac, external and internal iliac, and presacral lymph nodes. The planning target volume (PTV) was generated by using 7 mm uniform expansion of the CTV. OARs were contoured on the full bladder scan using RTOG guideline and including bladder, bowel cavity, rectum, femoral heads, and other normal tissues.^20^

All plans were generated by a senior dosimetrist. Four‐field CRT plans were based on anatomical borders. These borders were: Superior–L5/S1; Inferior–Bottom of the obturator foramen; Lateral–2 cm on the pelvic brim, with adjustments based on vessel contours; Anterior–5 mm anterior to pubic symphysis with adjustments based on vessel contours; and Posterior–S2/S3. Seven equally spaced coplanar fields were used for the IMRT plans. The gantry angles were as follows: 0, 51, 102, 153, 204, 255, and 306. Two‐arc VMAT plans were optimized with a leaf motion of 0.46 cm/deg and a final arc space degree of 4.

The prescription dose was 45 Gy for PTV at 1.8 Gy per fraction. The planning goal for both VMAT and IMRT was to obtain 95% of the prescribed dose to cover 98% of the PTV and not to exceed 110% as maximum dose. For the OARs of rectum, bladder and small bowel, the dose received by 2% of the tissue volume (D2%) defined as the maximum dose was limit to 45 Gy. The complementary constraints V40 Gy (the volume receiving 40 Gy of radiation) were < 40% for the rectum, < 50% for the bladder, < 25% for the small bowel, and < 5% for the femoral heads.

### 2.C Dosimetric evaluation and comparison

Quantitative evaluation of plans was performed by means of standard dose–volume histogram (DVH). For PTV, the values of D98% and D2% (dose received by the 98% and 2% of the volume) were defined as metrics for minimum and maximum doses and consequently reported. V95% (the volume receiving at least 95% of the prescribed dose) was reported as the target coverage. Homogeneity index (HI) was evaluated as the difference between the dose to 1% (*D*1) and 99% (*D*99) of PTV divided by the prescription dose (*Dp*),^21^

(1) $$HI=\frac{D1-D99}{Dp}\times 100\%$$

Conformity index (CI)^22^ and conformation number (CN)^23^ were also calculated for PTV:

(2) $$\text{CI}=\frac{V_{T,\mathrm{Pi}}}{V_{\mathrm{Pi}}}$$

(3) $$\text{CN}=\frac{V_{T,\mathrm{Pi}}}{V_{T}}\times \frac{V_{T,\mathrm{Pi}}}{V_{\mathrm{Pi}}}$$

where V_T,Pi_ is the volume of target that is covered by the prescription dose, V_T_ is the volume of target, and V_Pi_ is the volume of the body that is covered by the prescription isodose. The maximum value of CI is 1, corresponding to a perfect coverage of PTV. The CN is the complementary information to compensate for the defects of target coverage and CI. CN can take values between 0 and 1, where an ideal dose distribution would have a CN value of 1.

For OARs and health tissues, the analysis included the mean dose and a set of appropriate V_X_ and D_Y_ values. MU, delivery time difference and gamma passing rate for IMRT and VMAT were also evaluated and compared. VMAT and IMRT QA were performed using a 3D diode array ArcCHECK (Model 1220) and (SNC Patient v. 6.2.1; Sun Nuclear Corporation, Melbourne, FL, USA) with a global gamma passing criteria of 3%/3 mm and 10% lower dose threshold.

### 2.D Statistical analysis

Comparisons of dosimetric and nondosimetric indices among plans with different treatment modalities were analyzed with one‐way analysis of variance method. All statistical analysis was conducted with SPSS 17.0 software. Differences were considered statistically significant if *P* < 0.05.

## 3 Results

**Figure 1:**
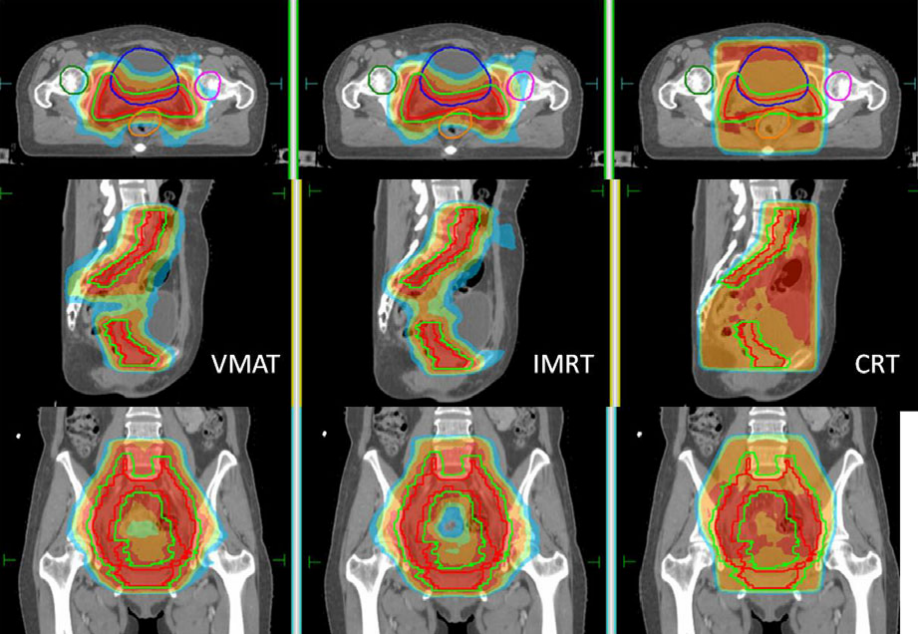
Typical dose distribution of CRT, IMRT, and VMAT plans for one postoperative cervical cancer patients.

**Figure 2:**
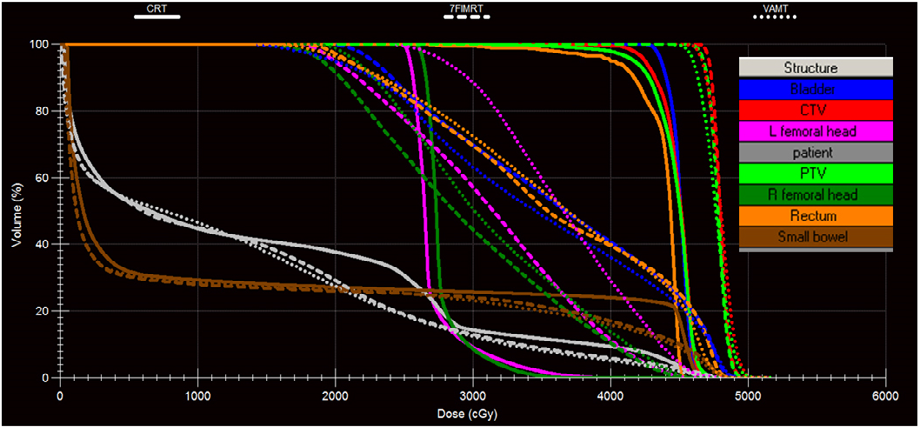
Dose‐volume histogram comparison among CRT, IMRT, and VMAT plans for one postoperative cervical cancer patient.

The patient characteristics were presented in (Table 1). The median age of these patients was 56 years (range 28–69 years). Figure 1 shows a typical dose distribution of one patient for CRT, IMRT and VMAT plans. Figure 2 shows a typical DVH of one patient for comparison among CRT, IMRT, and VMAT plans.

**Table 1:** Patients characteristics and target volumes of 15 postoperative cervical cancer patients

Patients	Age (*y*)	TNM stage	CTV (cm^3^)	PTV (cm^3^)
1	53	IB1	558.8	1064.6
2	42	IIa	546.8	864.2
3	69	IIA1	442.4	742.8
4	63	IIA1	567.1	1008.1
5	52	IB1	461.7	860.4
6	59	IB1	573.4	1059.1
7	61	IB1	674.2	1206.1
8	60	IIA2	418.3	846.5
9	56	IB1	458.8	876.4
10	64	IB1	415.6	847.8
11	63	IB1	482.1	930.2
12	46	IB2	422.6	816.6
13	37	IB1	390.3	767.5
14	28	IIA2	402.8	808.5
15	55	IB2	498.7	929.1

Detailed dosimetric differences on target coverage and OAR sparing were presented in (Table 2). Target coverage presented with V95% were 88.9% ± 3.8%, 99.9% ± 0.07%, and 99.9% ± 0.1% for CRT, IMRT, and VMAT, respectively. VMAT and IMRT achieved a significant higher CI and CN compared with CRT with CIs of 0.37 ± 0.07, 0.55 ± 0.04, 0.61 ± 0.04 and CNs of 0.33 ± 0.06, 0.55 ± 0.04, and 0.60 ± 0.04 for CRT, IMRT, and VMAT, respectively.

**Table 2:** Detailed dosimetric comparison among CRT, IMRT, and VMAT

	CRT	IMRT	VMAT	*P*		
				CRT vs. IMRT	CRT vs. VMAT	IMRT vs. VMAT
PTV						
Dmax (cGy)	4788.5 ± 74.2	5128.1 ± 50.6	5189.5 ± 32.0	<0.001	<0.001	0.01
Dmean (cGy)	4446.0 ± 42.6	4748.7 ± 34.3	4743.8 ± 24.3	<0.001	<0.001	0.92
V95 (%)	88.9 ± 3.8	99.9 ± 0.07	99.9 ± 0.1	<0.001	<0.001	0.99
D2 (cGy)	4650.8 ± 48.9	4907.0 ± 47.9	4962.2 ± 22.5	<0.001	<0.001	0.002
D98 (cGy)	3822.9 ± 287.1	4587.0 ± 45.3	4494.9 ± 51.0	<0.001	<0.001	0.31
HI	0.19 ± 0.07	0.07 ± 0.01	0.10 ± 0.01	<0.001	<0.001	0.09
CI	0.37 ± 0.07	0.55 ± 0.04	0.61 ± 0.04	<0.001	<0.001	0.01
CN	0.33 ± 0.06	0.55 ± 0.04	0.60 ± 0.04	<0.001	<0.001	0.008
Bladder						
Dmean (cGy)	4418.6 ± 169.0	3595.7 ± 135.1	3476.3 ± 188.3	<0.001	<0.001	0.13
V45 (%)	66.2 ± 15.9	21.8 ± 4.5	19.2 ± 4.2	<0.001	<0.001	0.75
V40 (%)	92.8 ± 10.1	39.4 ± 5.3	37.3 ± 6.3	<0.001	<0.001	0.73
Rectum						
Dmean (cGy)	4423.9 ± 81.7	3694.4 ± 93.9	3730.7 ± 101.0	<0.001	<0.001	0.53
V45 (%)	42.8 ± 28.0	24.6 ± 6.0	18.4 ± 6.6	0.01	0.001	0.58
V40 (%)	96.1 ± 2.9	44.3 ± 5.0	44.5 ± 4.8	<0.001	<0.001	0.99
Small bowel						
Dmax (cGy)	4487.8 ± 793.3	4943.2 ± 63.3	5013.9 ± 58.0	0.03	0.009	0.91
Dmean (cGy)	1578.1 ± 576.5	1404.6 ± 461.5	1328.2 ± 462.9	0.62	0.37	0.91
V45 (%)	8.8 ± 8.6	6.5 ± 3.3	5.0 ± 2.6	0.51	0.15	0.72
V40 (%)	19.8 ± 11.0	12.3 ± 5.0	10.4 ± 4.8	0.02	0.004	0.77
V30 (%)	25.9 ± 13.7	23.1 ± 9.3	20.2 ± 9.0	0.76	0.34	0.76
Left femoral head						
Dmean (cGy)	2718.0 ± 47.2	2862.8 ± 315.9	3158.2 ± 438.1	0.42	0.001	0.04
V45 (%)	0	1.5 ± 1.8	4.5 ± 4.5	0.32	<0.001	0.02
V40 (%)	0.07 ± 0.2	9.9 ± 7.1	17.0 ± 10.6	0.002	<0.001	0.03
Right femoral head						
Dmean (cGy)	2764.8 ± 85.2	2779.1 ± 348.2	3064.4 ± 233.3	0.99	0.005	0.008
V45 (%)	0	1.3 ± 2.0	1.9 ± 2.4	0.13	0.02	0.72
V40 (%)	0.5 ± 1.2	9.2 ± 7.2	12.7 ± 8.0	0.001	<0.001	0.27
Health tissues						
V5 (%)	52.2 ± 8.9	51.7 ± 8.0	53.0 ± 7.9	0.98	0.96	0.90
V10 (%)	43.5 ± 8.6	44.1 ± 7.1	45.2 ± 7.1	0.98	0.83	0.92
V15 (%)	39.8 ± 8.2	37.6 ± 6.2	35.1 ± 5.8	0.66	0.15	0.56

Significant differences on CRT vs. IMRT and CRT vs. VMAT were observed for bladder and rectum sparing. IMRT and VMAT decreased the Dmean, V45 and V30 of small bowel compared with CRT, although the differences were of no statistical significance. However, IMRT (12.3 ± 5.0 vs. 19.8 ± 11.0, *P *= 0.02) and VMAT (10.4 ± 4.8 vs. 19.8 ± 11.0, *P* = 0.004) decreased V40 of small bowel significantly compared with CRT. IMRT and VMAT increased the maximum dose to small bowel and the irradiation dose to femoral heads compared with CRT. For heath tissues, there was no significant difference observed among these three modalities.

The average MUs for CRT, IMRT and VMAT were 232.4 ± 5.6, 957.2 ± 47.8, and 852.4 ± 73.8, respectively. The treatment delivery time for IMRT and VMAT were 9.4 ± 0.6 and 3.3 ± 0.1 minutes, respectively. The average gamma passing rate for IMRT and VMAT at 3%/3 mm criteria were 95.2% ± 1.1% and 94.7% ± 0.9%, respectively.

## 4 Discussion

The dosimetric advantages of VMAT in the treatment of postoperative cervical cancer patients were investigated by comparing directly with CRT and IMRT in this study. VMAT and IMRT increased the target coverage, conformity significantly compared with CRT. VMAT and IMRT also increased the protection on bladder, rectum, and small bowel compared with CRT. VMAT achieved a better dose conformity, less MU and shorter delivery time than IMRT. No significant difference on OAR sparing was observed between IMRT and VMAT.

It had been reported on many cancer sites that IMRT and VMAT can generate non‐uniform fields to achieve better planning target volume coverage, while decreasing unnecessary radiation exposure to normal organs.^24^ In this study, about 99.9% PTV was covered by the prescription dose with IMRT and VMAT compared with 88.9% of CRT. These were similar to the reported 98.1% PTV coverage with intensity‐modulated whole pelvic radiotherapy in women with gynecological malignancies.^25^ Consistently, VMAT and IMRT also increased the CI and CN of target significantly compared with CRT.

In this study, the irradiated volume and dose of bladder and rectum presented by Dmean, V40 and V45 were greatly decreased in IMRT and VMAT compared with those in CRT. This was consistent with the report that the pooled average irradiated volumes of IMRT were lower than that of CRT for rectums that received ≥ 30 Gy, 40 Gy, and 45 Gy.^26^ In this study, although IMRT and VMAT decreased the Dmean, V45 and V30 of small bowel compared with CRT, only V40 showed a statistical significance (*P* = 0.02 for IMRT vs. CRT, and *P* = 0.004 for VMAT vs. CRT). This was slightly different from the previously reported results that IMRT delivered remarkably less average irradiated volumes of small bowel for doses of > 30 Gy and > 45 Gy than CRT.^27^ This may be caused by the OAR volume variation due to its unstable shape in an abdominal cavity, may be also due to different bladder filling condition applied for different patients and centers. IMRT and VMAT actually increased the maximum dose to small bowel resulted from the increased PTV maximum dose as a cost of better conformity. IMRT and VMAT also increased the dose irradiated to the left and right femoral head compared with CRT. This was expectable since CRT can block the femoral head manually. For normal tissue sparing, there was no significant difference observed between VMAT, IMRT, and CRT.

VMAT had a higher maximum dose to PTV as presented by Dmax and D2 compared with IMRT for achieving a better conformity. There was no other significant difference on target coverage between IMRT and VMAT observed. Similarly, VMAT irradiated more dose to the left and right femoral heads compared with IMRT. VMAT actually irradiated less dose to bladder, rectum, and small bowel compared with IMRT, although the differences were not statistically significant. There was no other significant difference on OAR sparing observed between IMRT and VMAT in this study. VMAT irradiated the largest volume of health tissue with a lower dose (V5 and V10) and the least volume with a medium dose (V15) compared with IMRT and CRT, although these differences were of no statistical significance. This was different from the study of Cozzi et al, in which the authors stated that RapidArc showed significant improvements in OAR and healthy tissue sparing with uncompromised target coverage compared with conventional five‐field IMRT16. On the contrary, Sharfo et al stated that often reported increased plan quality for VMAT compared to IMRT has not been observed for cervical cancer in their study.^17^ This differences indicated that the number of fields used for IMRT has a direct impact on the quality of the IMRT plan. VMAT decreased the MU and delivery time as reported in the previous studies.

## 5 Conclusion

VMAT showed significant dosimetric advantages both on target coverage and OAR sparing compared with CRT in the treatment of postoperative cervical cancer. However, no significant difference between IMRT and VMAT was observed except for slightly better dose conformity, slightly less MU and significant shorter delivery time achieved for VMAT.

## Conflict of interest

The authors declare that they have no competing interest.
